# Regional Analysis of Coccidioidomycosis Incidence — California, 2000–2018

**DOI:** 10.15585/mmwr.mm6948a4

**Published:** 2020-12-04

**Authors:** Gail L. Sondermeyer Cooksey, Alyssa Nguyen, Duc Vugia, Seema Jain

**Affiliations:** 1 Infectious Diseases Branch, Center for Infectious Diseases, California Department of Public Health.

Coccidioidomycosis (Valley fever) is an infection caused by the soil-dwelling fungus *Coccidioides* spp., which usually manifests as a mild self-limited respiratory illness or pneumonia but can result in severe disseminated disease and, rarely, death (*1*,*2*). In California, coccidioidomycosis incidence increased nearly 800% from 2000 (2.4 cases per 100,000 population) to 2018 (18.8) (*2*–*4*). The California Department of Public Health (CDPH) reports statewide and county-level coccidioidomycosis incidence annually; however, a comprehensive regional analysis has not been conducted. Using California coccidioidomycosis surveillance data during 2000–2018, age-adjusted incidence rates were calculated, and coccidioidomycosis epidemiology was described in six regions. During 2000–2018, a total of 65,438 coccidioidomycosis cases were reported in California; median statewide annual incidence was 7.9 per 100,000 population and varied by region from 1.1 in Northern and Eastern California to 90.6 in the Southern San Joaquin Valley, with the largest increase (15-fold) occurring in the Northern San Joaquin Valley. When analyzing demographic data, which was available for >99% of cases for sex and age and 59% of cases for race/ethnicity, median annual incidence was high among males (10.2) and Black persons (9.0) consistently across all regions; however, incidence varied among Hispanics and adults aged 40–59 years by region. Tracking these surveillance data at the regional level reinforced understanding of where and among what demographic groups coccidioidomycosis rates have been highest and revealed where rates are increasing most dramatically. The results of this analysis influenced the planning of a statewide coccidioidomycosis awareness campaign so that the messaging, including social media and TV and radio segments, focused not only on the general population in the areas with the highest rates, but also in areas where coccidioidomycosis is increasing at the fastest rates and with messaging targeted to groups at highest risk in those areas. 

Coccidioidomycosis incidence is highest in the southwestern United States, with approximately 97% of cases reported from Arizona and California (*1*,*2*). Environmental data on where *Coccidioides* exists in the soil are limited; therefore, understanding the geographic risk for infection is largely based on human surveillance data. In California, from 1995 to 2011, annual coccidioidomycosis incidence fluctuated from a low of 1.9 cases per 100,000 in 1997 to a high of 13.9 in 2011. In 2014, incidence declined to 6.0, then increased to 19.3 (>200% increase) in 2017 and remained high (18.8) in 2018. Over the last 18 years, median annual incidence was less than two per 100,000 population in two thirds (39 of 58) of California counties, although it ranged from 13 to 182 in seven counties located in the Central Valley and Central Coast (*3*,*4*). Incidence has been consistently high in six counties in the Southern San Joaquin Valley (Fresno, Kern, Kings, Madera, and Tulare counties) and Central Coast (San Luis Obispo County) regions (*3*,*4*). Coccidioidomycosis has been reportable in California since 1995 and laboratory-reportable since 2014 (*2*–*4*). From 1995 to 2018, the 61 California local health jurisdictions reported data to CDPH using the Council of State and Territorial Epidemiologists case definition for coccidioidomycosis, which includes clinical and laboratory criteria* (*5*). Because of the high disease incidence and resources needed to confirm symptoms, some local health jurisdictions recently transitioned to a laboratory-only case definition for coccidioidomycosis; the final determination of a confirmed case is decided by the local health jurisdiction. For this analysis, regions were based on historic county-level coccidioidomycosis surveillance data and geographic environmental and climatic factors that might affect where *Coccidioides* could proliferate in California; counties were grouped into regions based on similar coccidioidomycosis incidence and environmental profiles (*3*–*6*). The six regions were the 1) Central Coast, 2) Northern and Eastern California, 3) Northern San Joaquin Valley, 4) Southern Coast, 5) Southern Inland, and 6) Southern San Joaquin Valley.

Using 2000–2018 California coccidioidomycosis surveillance data, age-adjusted annual incidence rates were calculated statewide and by region per 100,000 population using population data from the California Department of Finance derived from the 2000 U.S. Census.^†^ A confirmed case was defined as coccidioidomycosis in a California resident as determined by the local health jurisdiction. To describe recent incidence increases, rate ratios were calculated, including by region, to compare rates in 2000 and 2014 with those in 2018. The relative risk for coccidioidomycosis and corresponding 95% confidence intervals were calculated by year (continuous), sex, age group, race/ethnicity, and region using multivariable negative binomial regression (with statistical significance defined as p<0.05) to assess trends and demographic differences by region. This model was fit to the aggregated statewide data, both unadjusted and adjusted for region, and stratified by region. A multivariable analysis was not conducted for the Northern and Eastern California region because of insufficient power. Because specific race/ethnicity data were missing (“Other” or “Unknown”) for 41% of reported cases, the multivariable model was restricted to cases with complete data. All analyses were performed using SAS (version 9.4; SAS Institute).

During 2000–2018, a total of 65,438 coccidioidomycosis cases were reported in California, with a median age-adjusted annual incidence of 7.9 per 100,000 population. Annual age-adjusted statewide incidence was lowest in 2000 (2.4), peaked in 2017 (18.9), and decreased slightly in 2018 (18.3) (Supplementary Table, https://stacks.cdc.gov/view/cdc/97708). The highest median annual incidences were in the Southern San Joaquin Valley (90.6), Central Coast (9.7), and Northern San Joaquin Valley (5.6); and lowest in the Southern Coast (2.7), Southern Inland (2.2) and Northern and Eastern California (1.1) (Table 1). In all regions, incidence was higher among males than among females, among persons aged ≥40 years than among those aged <40 years; and, for cases where race/ethnicity data were present, among Black persons than among other racial/ethnic groups.

**TABLE 1:** Demographic characteristics of persons with confirmed coccidioidomycosis cases (n = 65,438), by region of residence* — California, 2000–2018

Characteristic	No. (%) [incidence]^†^						
	California	Regions*					
		Southern San Joaquin Valley	Central Coast	Northern San Joaquin Valley	Southern Coast	Southern Inland	Northern and Eastern California
**Overall**	**65,438 (100) [7.9]**	**42,198 (100) [90.6]**	**5,312 (100) [9.7]**	**2,890 (100) [5.6]**	**9,999 (100) [2.7]**	**1,964 (100) [2.2]**	**2,772 (100) [1.1]**
**Sex**							
Male	41,902 (64.6) [10.2]	26,776 (63.8) [110.9]	3,569 (67.2) [13.4]	1,901 (66.1) [8.2]	6,267 (62.9) [3.4]	1,400 (71.3) [3.3]	1,989 (72.3) [1.7]
Female	22,943 (35.4) [5.5]	15,204 (36.2) [60.4]	1,740 (32.8) [5.5]	977 (33.9) [3.2]	3,698 (37.1) [1.9]	563 (28.7) [1.2]	761 (27.7) [0.5]
**Age group (yrs)**							
0–19	7,304 (11.2) [3.1]	6,009 (14.2) [32.5]	415 (7.8) [2.4]	229 (7.9) [1.4]	441 (4.4) [0.5]	88 (4.5) [0.2]	122 (4.4) [0.2]
20–39	21,147 (32.5) [9.3]	15,743 (37.3) [104.7]	1,366 (25.7) [9.9]	633 (21.9) [5.7]	2,253 (22.5) [2.1]	481 (24.5) [1.7]	671 (24.2) [0.2]
40–59	23,583 (36.2) [10.8]	14,485 (34.3) [123.0]	2,129 (40.1) [15.4]	1,205 (41.7) [11.2]	3,828 (38.3) [3.7]	809 (41.2) [3.3]	1,127 (40.7) [1.6]
≥60	13,101 (20.1) [7.9]	5,961 (14.1) [70.8]	1,402 (26.4) [14.9]	823 (28.5) [8.0]	3,477 (34.8) [5.6]	586 (29.8) [4.0]	852 (30.7) [2.0]
**Race/Ethnicity**							
White	14,024 (33.0) [4.3]	7,072 (27.5) [39.2]	2,108 (51.2) [8.7]	518 (34.5) [2.8]	3,273 (39.2) [1.9]	509 (36.6) [1.3]	544 (39.1) [0.3]
Black	4,062 (9.6) [9.0]	2,378 (9.2) [123.8]	235 (5.7) [19.2]	111 (7.4) [8.1]	956 (11.4) [3.7]	184 (13.2) [2.8]	198 (14.2) [1.2]
Hispanic	19,484 (45.9) [7.1]	13,874 (54.0) [60.0]	1,420 (34.5) [8.2]	569 (37.9) [5.3]	2,787 (33.4) [2.1]	516 (37.1) [1.7]	318 (22.8) [0.6]
API	2,763 (6.5) [2.4]	1,122 (4.4) [38.6]	164 (4.0) [7.1]	185 (12.3) [7.4]	962 (11.5) [1.6]	92 (6.6) [1.7]	238 (17.1) [0.3]

**Abbreviation:** API = Asian/Pacific Islander.

* Southern San Joaquin Valley (Fresno, Kern, Kings, Madera, and Tulare counties), Central Coast (Monterey, San Luis Obispo, Santa Barbara, and Ventura counties), Northern San Joaquin Valley (Merced, San Benito, San Joaquin, and Stanislaus counties), Southern Coast (Los Angeles, Orange, and San Diego counties), Southern Inland (Imperial, Riverside, and San Bernardino counties), and Northern and Eastern California (all other California counties).

^†^ Cases per 100,000 population; median annual incidence from 2000 to 2018 was age-adjusted to the United States 2000 standard population.

During the study period, incidence increased statewide and by region (Figure). When comparing the incidence in 2018 statewide with that in 2000, the rate ratio was 7.5, with highest rate ratios in the Northern San Joaquin Valley (15.3) and Southern Coast (8.8). Comparing incidence in 2018 with that in 2014, the highest rate ratio was in the Central Coast (8.1) and ranged from 2.5–3.3 in all other regions.

**FIGURE:**
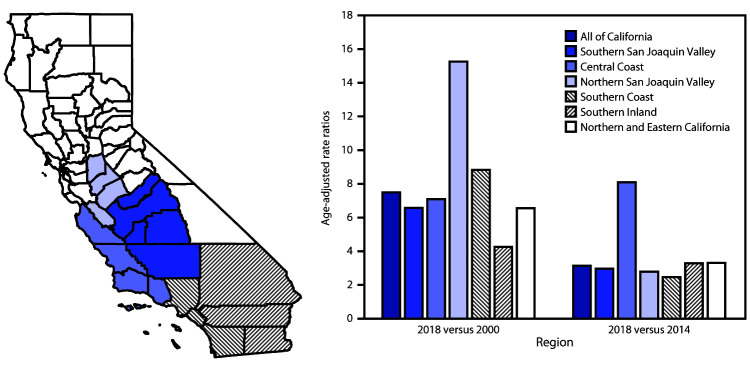
Ratios of age-adjusted^†^ annual coccidioidomycosis incidence, by region* — California, 2018 versus 2000 and 2018 versus 2014 * Southern San Joaquin Valley (Fresno, Kern, Kings, Madera, and Tulare counties), Central Coast (Monterey, San Luis Obispo, Santa Barbara, and Ventura counties), Northern San Joaquin Valley (Merced, San Benito, San Joaquin, and Stanislaus counties), Southern Coast (Los Angeles, Orange, and San Diego counties), Southern Inland (Imperial, Riverside, and San Bernardino counties), and Northern and Eastern California (all other California counties). ^†^ All rates were age-adjusted to the United States 2000 standard population.

In statewide and regional multivariable models, which included only cases with complete demographic data, the relative risk (RR) for coccidioidomycosis among males compared with that among females ranged from 1.91 (Southern Coast) to 2.86 (Southern Inland) (Table 2). Compared with cases in persons aged <20 years, the RR was highest among adults aged 40–59 years (RR = 4.03) in the statewide unadjusted model. After adjusting for region, the RR was highest among adults aged ≥60 years (RR = 5.92). When stratified by region, RR was highest among adults aged 40–59 years in the Southern San Joaquin Valley (3.89) and Central Coast (5.24); and highest among adults aged ≥60 years in all other regions (range = 7.37–12.56). In the statewide unadjusted model, RRs were similarly higher among Black (1.76) and Hispanic persons (1.81), compared with White persons. However, when adjusted for region, the RRs for Black and Hispanic persons were 2.13 and 1.21, respectively. When stratified by region, Hispanic persons were at significantly higher risk than were Whites in the Southern San Joaquin Valley (1.55) and the Northern San Joaquin Valley (1.41), whereas the RR for Black persons was significantly higher in all regions, ranging from 1.84 to 2.25.

**TABLE 2:** Multivariable regression models for risk for coccidioidomycosis not adjusted, adjusted, and stratified, by region — California, 2000–2018 (n = 40,264*)

Characteristic	Relative risk^†^ (95% CI)						
	California		Regions^§^				
	Not adjusted for region	Adjusted for region	Southern San Joaquin Valley	Central Coast	Northern San Joaquin Valley	Southern Coast	Southern Inland
**Year (continuous)^¶^**	1.07 (1.06–1.08)	1.08 (1.08–1.09)	1.05 (1.04–1.06)	1.08 (1.07–1.09)	1.10 (1.08–1.11)	1.12 (1.11–1.13)	1.04 (1.02–1.05)
**Sex (ref = female)**							
Male	2.12 (1.96–2.29)	2.20 (2.09–2.31)	1.95 (1.77–2.13)	2.32 (2.03–2.64)	2.28 (1.98–2.61)	1.91 (1.78–2.04)	2.86 (2.46–3.33)
**Age group (yrs) (ref = 0–19)**							
20–39	3.04 (2.73–3.39)	3.41 (3.15–3.69)	3.18 (2.79–3.61)	3.00 (2.45–3.68)	3.19 (2.51–4.07)	4.57 (4.00–5.23)	5.34 (3.97–7.18)
40–59	4.03 (3.61–4.50)	5.60 (5.18–6.06)	3.89 (3.41–4.43)	5.24 (4.29–6.39)	6.59 (5.24–8.30)	8.39 (7.35–9.56)	10.28 (7.71–13.70)
≥60	3.77 (3.37–4.21)	5.92 (5.45–6.42)	2.98 (2.60–3.41)	4.64 (3.77–5.71)	7.37 (5.79–9.38)	11.73 (10.27–13.40)	12.56 (9.34–16.89)
**Race/Ethnicity (ref = White)**							
Black	1.76 (1.59–1.96)	2.13 (1.98–2.29)	2.21 (1.94–2.51)	2.22 (1.81–2.72)	2.09 (1.65–2.63)	1.84 (1.67–2.04)	2.25 (1.83–2.76)
Hispanic	1.81 (1.63–2.00)	1.21 (1.14–1.29)	1.55 (1.37–1.74)	0.92 (0.79–1.07)	1.41 (1.20–1.65)	0.98 (0.90–1.07)	1.07 (0.90–1.27)
API	0.61 (0.55–0.68)	0.98 (0.91–1.06)	1.07 (0.93–1.22)	0.68 (0.55–0.84)	1.72 (1.41–2.10)	0.81 (0.73–0.89)	1.29 (1.00–1.66)
**Region of residence (ref = Northern and Eastern California)**							
**Southern San Joaquin Valley**	N/A	90.39 (81.63–100.09)	N/A	N/A	N/A	N/A	N/A
**Central Coast**	N/A	16.12 (14.46–17.98)	N/A	N/A	N/A	N/A	N/A
**Northern San Joaquin Valley**	N/A	7.60 (6.76–8.55)	N/A	N/A	N/A	N/A	N/A
**Southern Coast**	N/A	3.55 (3.20–3.93)	N/A	N/A	N/A	N/A	N/A
**Southern Inland**	N/A	2.44 (2.16–2.74)	N/A	N/A	N/A	N/A	N/A

**Abbreviations:** API = Asian/Pacific Islander; CI = confidence interval; cont. = continuous variable; N/A = not applicable; ref = regression reference group.

* Multivariable models included only data from cases with complete sex, age, and race/ethnicity.

**^†^** Multivariable relative risk and associated 95% confidence intervals were calculated by negative binomial regression.

^§^ Southern San Joaquin Valley (Fresno, Kern, Kings, Madera, and Tulare counties), Central Coast (Monterey, San Luis Obispo, Santa Barbara, and Ventura counties), Northern San Joaquin Valley (Merced, San Benito, San Joaquin, and Stanislaus counties), Southern Coast (Los Angeles, Orange, and San Diego counties), Southern Inland (Imperial, Riverside, and San Bernardino counties), and Northern and Eastern California (all other California counties).

^¶^ Year was included in the model as a continuous variable.

## Discussion

The Southern San Joaquin Valley and Central Coast regions have the highest consistent coccidioidomycosis incidences in California, and the hot, dry climate and environment in these regions is known to be suitable for *Coccidioides* proliferation; predictive ecological niche modeling has indicated that *Coccidioides* could expand to other areas (*6*). Although increasing case counts in the Southern San Joaquin Valley have contributed most to the overall increases in statewide coccidioidomycosis incidence, these regional analyses indicate that the largest increases in incidence occurred outside the Southern San Joaquin Valley, particularly in the Northern San Joaquin Valley and Southern Coast, and, since 2014, in the Central Coast. During this time, coccidioidomycosis outbreaks were infrequently reported (approximately one or two per year) and would not have affected overall surveillance trends. Although these increases in previously lower-incidence regions could reflect expanding areas where *Coccidioides* can proliferate, they might also reflect regional changes in work or recreation travel patterns, testing and reporting practices, or population susceptibility. Outside of California, coccidioidomycosis incidence also increased during 2000–2011 in Arizona, which reports approximately 65% of national cases, and in other states, which report approximately 3% of national cases, after which incidence either decreased or remained stable in those areas (*2*,*7*).

Black persons and older adults are known to be at increased risk for severe coccidioidomycosis (i.e., hospitalization or disseminated disease) (*8*) and were consistently found to be at higher risk for coccidioidomycosis in all California regions; the reasons for this are not completely understood but might include host characteristics (e.g., genetic factors and prevalence of comorbidities) and societal factors, (e.g., access to care and socioeconomic status) (*8,**9*). The risk for coccidioidomycosis in males has consistently been higher than that for females over time and by region, possibly related to exposure from outdoor work or recreational activities (*3*,*4*). In contrast, coccidioidomycosis risk in Hispanics compared with that in Whites and in adults aged 40–59 years compared with that in persons aged <20 years varied by region, suggesting that infection in these groups might be more influenced by environmental exposures in certain regions, possibly related to work or recreational outdoor activities, particularly those involving dirt or dust. The majority of coccidioidomycosis outbreaks in California have occurred in high-incidence regions and have been associated with dirt-disturbing work settings, including construction, military, archeologic sites, and correctional institutions, where high attack rates have been seen even among relatively young, healthy populations (*10*). Further research is needed to better delineate the factors associated with increased risk in these groups in some but not all regions.

The findings in this report are subject to at least four limitations. First, the data were limited by the quality of provider and laboratory-based coccidioidomycosis reporting and local health jurisdiction ability to review and confirm cases; these results might mostly reflect patients with moderate or severe coccidioidomycosis, including those at higher risk for severe disease such as Black persons and older adults, because mild illness is less likely to be diagnosed and reported. Second, cases were reported based on patients’ residential address, which might not reflect the exposure area. Third, although the most common types of diagnostic test used for coccidioidomycosis during this period have not changed, it is not known whether and how testing practices might have changed and how that might have affected incidence in various regions or among certain groups. Finally, 41% of cases had an unknown or other race-ethnicity; therefore, regional estimates by race/ethnicity might be biased by the counties where reporting was more complete.

In a large, diverse state, such as California, analysis of coccidioidomycosis surveillance data at a regional level improved the understanding of disease trends, emergence, and epidemiology and informed efforts to improve public and provider awareness, directing messaging to areas with increasing trends that are outside of the typical high incidence regions. Currently, no effective methods are known for primary prevention of coccidioidomycosis (e.g., a vaccine); therefore, widespread awareness is important to prompt early diagnosis, proper management, possible antifungal treatment, and better outcomes. The results of these analyses helped focus statewide and regional outreach efforts, including targeted social media messages and the distribution of awareness resources to communities at risk in areas with high or increasing incidence and assisted in identifying the most affected demographic and occupational groups to target within specific regions.

**Summary**

**What is already known about this topic?**

Coccidioidomycosis incidence increased in California from 2000 to 2018 and was higher among males, adults aged ≥40 years, Black persons, and residents of Central California.

**What is added by this report?**

In the first regional analysis of coccidioidomycosis in California, risk was consistently high across California regions among males and Black persons yet varied by region among different age groups and Hispanic ethnicity. Incidence was highest in the Southern San Joaquin Valley, and the largest increase from 2000 to 2018 occurred in the Northern San Joaquin Valley.

**What are the implications for public health practice?**

Routine regional analysis of coccidioidomycosis data should be performed to better understand where increases are occurring and whether risk by demographic groups varies, and these results should be used to better target and tailor outreach messaging.

## Footnotes

* https://wwwn.cdc.gov/nndss/conditions/coccidioidomycosis/case-definition/2011.
